# Sevoflurane protects cardiomyocytes against hypoxia/reperfusion injury via LINC01133/miR-30a-5p axis

**DOI:** 10.1042/BSR20200713

**Published:** 2020-11-26

**Authors:** Zhenyi Yu, Qiusheng Ren, Shenghui Yu, Xiang Gao

**Affiliations:** Department of Anesthesiology, Ningbo Yinzhou People's Hospital, Ningbo, Zhejiang Province, China

**Keywords:** hypoxia/reperfusion injury, LINC01133/miR-30a-5p axis, Sevoflurane

## Abstract

Previous studies failed to elucidate the detailed mechanisms of anesthetic preconditioning as a protective approach against ischemic/reperfusion (I/R) injury in cells. The present study mainly centered on discovering the mechanisms of Sevoflurane (Sev) in preventing cardiomyocytes against I/R injury. Human cardiomyocyte AC16 cell line was used to simulate I/R injury based on a hypoxia/reperfusion (H/R) model. After Sev treatment, cell viability and apoptosis were detected by MTT assay and flow cytometry, respectively. Lactate dehydrogenase (LDH) content was measured using an LDH Detection Kit. Relative mRNA and protein expressions of LINC01133, miR-30a-5p and apoptosis-related proteins were detected using quantitative real-time polymerase chain reaction (qRT-PCR) and Western blot as needed. Target gene of miR-30a-5p and their potential binding sites were predicted using Starbase and confirmed by dual-luciferase reporter assay. Cell behaviors were assessed again after miR-30a-5p and LINC01133 transfection. Sev could improve cell viability, reduce LDH leakage, and down-regulate the expressions of apoptosis-related proteins (Bax, cleaved caspase-3 and cleaved caspase-9) and LINC01133 as well as up-regulate miR-30a-5p and Bcl-2 expressions in H/R cells. MiR-30a-5p was the target of LINC01133, and up-regulating miR-30a-5p enhanced the effects of Sev in H/R cells, with a suppression on H/R-induced activation of the p53 signaling pathway. However, up-regulating LINC01133 reversed the enhancing effects of miR-30a-5p on Sev pretreatment in H/R cells. Sev could protect cardiomyocytes against H/R injury through the miR-30a-5p/LINC01133 axis, which may provide a possible therapeutic method for curing cardiovascular I/R injury.

## Introduction

With more than 80% of cases of death in low-income and middle-income countries, cardiovascular diseases (CVDs) are the leading causes of death around the world [1]. Among them, ischemia–reperfusion injury (I/R Injury) has been found in patients who suffered from myocardial ischemia and received treatment [2]. And it is a critical condition for which researchers are required to control damages on the cells and prevent organs from dysfunction [3]. Anesthetic preconditioning (APC), a cell protection mechanism, has been well-established for its benefits on cardiomyocyte protection as it has the capability to elicit a biphasic cardio-protection pattern [4]. In clinical practice, Sevoflurane (Sev) has been widely applied as an inhalational anesthetic and pretreatment with Sev has been found to induce ischemic tolerance *in vitro* and *in vivo* [5,6]; nevertheless, the underlying mechanisms for the protective effect of Sev pretreatment on cardiomyocytes remained to be further discovered and discussed.

Recently, it has been reported in many studies that non-coding RNAs (ncRNAs) played a critical role in I/R injury, and meantime, long non-coding RNAs (lncRNAs) have also been unveiled to have cellular functions, and, according to accumulating evidence, play vital roles in myocardial I/R injury [7]. For example, inhibition of lncRNA XIST, which exerts an inhibitory effect on autophagy and a regulatory effect on SOCS2, could relieve myocardial I/R injury via targeting miR-133a [8]. In addition, lncRNA H19 could alleviate myocardial I/R injury via suppressing the mitochondrial apoptosis mediated by miR-877-3p/Bcl-2 [9]. Long intergenic non-protein coding RNA 01133 (LINC01133) inhibits GC progression and metastasis by regulating miR-106a-3p and the Wnt/β-catenin pathway [10]. LINC01133 expression was significantly down-regulated in breast cancer samples and was associated with the progression and poor prognosis of breast cancer [11]. However, the relationship between lncRNA LINC01133 and ischemic/reperfusion has been rarely discussed. Apart from lncRNAs, microRNAs (miRNAs) have also been extensively studied in research on cardiovascular diseases [12]. As shown in Li et al.’s research, Salvianolic acid B-induced miR-30a up-regulation exerts a protective effect on cardiac myocytes against I/R injury [13]. Also, the study by Xu et al. indicated that exosome-carried miR-30a inhibitor could inhibit myocardial apoptosis in rats with I/R injury [14]. These findings imply that miR-30a plays a vital role in CVD development and progression. However, its role in the underlying molecular mechanisms of Sevoflurane pretreatment in cardiomyocyte protection is yet to be uncovered.

Since hypoxia/reperfusion (H/R) cellular model has been used to simulate I/R injury *in vitro* [15], our study mainly focused on investigating the molecular mechanisms via which LINC01133/miR-30a-5p played roles in myocardial I/R injury prevention, with the hope to find a possible treatment for cardiovascular I/R injury.

## Materials and methods

### Cell culture and treatment

Human cardiomyocytes cell line AC16 was obtained from Merck (Darmstadt, Germany) and was cultured in Dulbecco’s modified eagle’s medium (DMEM; Invitrogen, Carlsbad, CA, U.S.A.) supplemented with 10% fetal bovine serum (FBS; Invitrogen, U.S.A.) and 1% penicillin-streptomycin (HyClone, Logan, UT, U.S.A.) in a humidified incubator at 37°C with 5% CO_2_.

I/R injury in the cells was simulated based on an *in vitro* (H/R) model [15]. AC16 cells used in our experiments were divided into the groups as follows: Control, H/R, and Sevoflurane (Sev). Normal AC16 cells were used as the Control group and were treated as previously described. For the H/R group, AC16 cells were cultured in an incubator with 95% N_2_ and 5% CO_2_ for 6 h; after the culture medium was subsequently replaced with a fresh 10%-FBS medium, the cells were cultured with 5% CO_2_ for another 6 h. For the Sev group, AC16 cells received the same treatment as the H/R group, except that they were pre-cultured in 5% CO_2_ with 2% Sevoflurane (Sigma-Aldrich, St Louis, MO, U.S.A.) for 1 h before culture in an incubator [16].

### Cell transfection

MiR-30a-5p mimic and mimic control were obtained from Sigma-Aldrich Corp. (St Louis, MO, U.S.A.), and small interfering RNA for lncRNA LINC01133 (si LINC01133) was synthesized by RiboBio (Guangzhou, China). pcDNA3.1 plasmid (NC) and LINC01133-pcDNA3.1 plasmid were bought from Invitrogen (CA, U.S.A.). Twenty pmol of miR-30a-5p mimic, mimic control or small interfering RNA for LINC01133, or 2 µg of pcDNA3.1 plasmid or LINC01133-pcDNA3.1 plasmid were then transfected into AC16 cells (6 × 10^4^ cells/well in 12-well plates) with Lipofectamine 3000 reagent (Thermo Fisher Scientific, Waltham, MA, U.S.A.) after cells reached 50–80% confluence. Mimic, mimic control or siRNA was mixed with non-serum RPMI-1640 medium and Lipofectamine 3000 reagent, and then incubated with cells. After transfection for 48 h, cells were harvested for subsequent studies. Transfection sequences were listed in Table 1.

**Table 1:** Sequence for transfection

Gene	Sequences
siLINC01133 sense oligo	5′-GGUUGCAAUGCAAGUUCUAUA-3′
siLINC01133 antisense oligo	5′-UAGAACUUGCAUUGCAACCGG-3′
miR-30a-5p mimic	5′-UGUAAACAUCCUCGACUGGAAG-3′
miR-30a-5p mimic control	5′-AAGGCAAGCUGACCCUGAAGU-3′

### MTT assay

Transfected AC16 cells, treated with Sevoflurane (Sev) for 1 h and H/R for 12 h, were seeded in 48-well plates (1 × 10^5^ cells/well). Then MTT assay was applied to detect cell viability. In brief, 10 μl of MTT reagent (catalog number: K017; Fine Biotech Co., Ltd., Wuhan, China) was added into cells. After incubation at 37°C for 4 h, the supernatant of cell culture was discarded, and the visible formazan crystals were dissolved in dimethyl sulfoxide (DMSO; 472301, Sigma-Aldrich Corp., St Louis, MO, U.S.A.). Then the absorbance value at 560 nm was recorded using a SpectraMax Plus 384 microplate reader (Molecular Devices, LLC., San Jose, CA, U.S.A.).

### Lactate dehydrogenase (LDH) leakage assay

Transfected AC16 cells, treated with Sev for 1 h and H/R for 12 h, were seeded in 96-well plates (2 × 10^4^ cells/well). Then, after collecting the cell supernatant, 60 μl of LDH detection solution from an LDH Detection Kit (catalog number: MK401; Takara Bio, Tokyo, Japan) was added to 150 μl of cell supernatant for further incubation at 37°C in the dark. About 30 min later, mass LDH leakage in the cell supernatant was measured at 490 nm with the LDH Detection Kit following the manuals of the producer.

### Flow cytometry

Cell apoptosis was detected using an Annexin V-FITC/R-Propidium (PI) apoptosis kit (catalog number: K101; BioVision, Milpitas, CA, U.S.A.) in accordance with the producer’s instructions. In short, transfected cells were harvested after treatment with Sev or H/R, and then washed with cold phosphate-buffered saline (PBS) twice, followed by treatment with Annexin V-FITC at room temperature for 15 min in the dark as well as co-treatment with PI for less than 1 h. Cell apoptosis was then analyzed with a flow cytometer (APO006; FLISP Serine Protease Detection Kits, Bio-Rad, U.S.A.).

### RNA isolation and quantitative real-time polymerase chain reaction (qRT-PCR)

Trizol reagent (Invitrogen, Madison, MI, U.S.A.) was used to extract total RNA based on the protocols of the producer. Then the extracted RNA was preserved in a **−**80°C refrigerator. A Nano Drop 2000 biological spectrometer (catalog number: ND-LITE-PR; Thermo Fisher Scientific, U.S.A.) was later employed to determine the RNA concentration. Next, 1 μg of the total RNA was used to synthesize cDNA with a First-strand cDNA Synthesis Kit (E6300L; New England Biolabs, Ipswich, MA, U.S.A.) following the producer’s instructions. Subsequently, a QRT-PCR experiment was conducted with an SYBR PremixEx Taq II kit (RR820L, TaKaRa, Japan) in AriaMx real-time PCR System (G8830A, Agilent, Santa Clara, CA, U.S.A.) under the conditions as follows: at 95°C for 10 min, followed by 40 cycles of at 95°C for 15 s and at 60°C for 1 min. For detecting miRNA expression, cDNAs were synthesized using TaqMan miRNA Assays (Applied Biosystems, Shanghai, China), and expressions of mRNAs were determined using M-MLV reverse transcriptase (BioTeke, Beijing, China) and normalized to that of small nuclear RNA U6. Primer sequences were listed in Table 2. GAPDH and U6 were used as internal references. The 2^−ΔΔCT^ calculation method was adopted to quantify relative gene expressions [17].

**Table 2:** Primers of qRT-PCR

Gene	Primers
LINC01133	
Forward	5′-GCAGAGCCATGGTACTGGAG-3′
Reverse	5′-AGGTTGCGGTGAACTGAGA-3′
miR-30a-5p	
Forward	5′-AGGTCGTATCCAGTGCAATTG-3′
Reverse	5′-GTCGTATCCAGTGCGTGTCG-3′
Bcl-2	
Forward	5′-CGCCCCTGCTATGGTTTAG-3′
Reverse	5′-GAGATTTCAGCTGCTCTTGGAC-3′
Bax	
Forward	5′-TCCTGCACCTGTTACGGTTT-3′
Reverse	5′-TGCTTCCAATTACTTTAATCCTTTTT-3′
GAPDH	
Forward	5′-TGCCAAATATGACATCAAGAA-3′
Reverse	5′-GGAGTGGGTGTCGTCGCTGTTG-3′
U6	
Forward	5′-CTCGCTTCGGCAGCACATATACT-3′
Reverse	5′-ACGCTTCACGAATTTGCGTGTC-3′

### Western blot

In our study, the expressions of apoptosis-related proteins (Bax, Bcl-2, cleaved caspase-3, cleaved caspase-9, p53 and p21) were detected by Western blot as previously described [18]. In detail, protein was lysed and extracted with RIPA buffer (R0010; Solarbio, Beijing, China) shortly post cell collection, and a bicinchoninic acid (BCA) protein kit (PC0020; Solarbio, China) was then used to measure the protein concentration. Protein sample lysates (30 μg) were subsequently electrophoresed by 12% sodium dodecyl sulfate-polyacrylamide gel electrophoresis (SDS-PAGE; P1200; Solarbio, China), and then were transferred into polyvinylidene fluoride (PVDF) membrane (FFP28; Beyotime, China). Then, after being blocked with non-fat milk (5%) for 2 h, the membrane was incubated with the primary antibodies: anti-Bcl-2 antibody (rabbit, 1:2000, ab196495, Abcam, Cambridge, U.K.), anti-Bax antibody (rabbit, 1:2000, ab32503, Abcam, U.K.), anti-cleaved caspase-3 antibody (rabbit, 1:500, ab49822, Abcam, U.K.), anti-cleaved caspase-9 antibody (mouse, 1:1000, sc-133109, Santa Cruz Biotechnology, Dallas, TX, U.S.A.), anti-p53 antibody (mouse, 1:1000, ab26, Abcam, U.K.), anti-p21 antibody (rabbit, 1:1000, ab109199, Abcam, U.K.) and anti-GAPDH antibody (rabbit, ab181602, 1:10000, Abcam, U.K.) at 4°C overnight, with GAPDH used as an internal control. Then, the membrane was incubated with the secondary horseradish peroxidase (HRP)-conjugated antibodies: goat anti-rabbit IgG H&L (HRP) (goat, 1:2000, ab205718, Abcam, U.K.) and goat anti-mouse IgG H&L (HRP) (goat, ab205719, 1:2000, Abcam) at room temperature for 1 h, and thereafter washed with tris-buffer saline tween (TBST) for three times. Protein band from the samples was analyzed with an enhanced chemiluminescence (ECL) kit (SW2020; Solarbio, China). Grey values of the protein bands were calculated using ImageJ (version 5.0; Bio-Rad, Hercules, CA, U.S.A.).

### Target gene prediction and dual-luciferase reporter assay

The target gene of miR-30a-5p and their potential binding sites were predicted using Starbase (http://www.starbase.sysu.edu.cn), which were subsequently confirmed using dual-luciferase reporter assay.

PMIR-REPORT Luciferase vector (catalog number: AM5795; Thermo Fisher Scientific, U.S.A.) that contained LINC01133 sequences (wild-type or mutated) was cloned into the pMirGLO reporter vector (Promega, Madison, WI, U.S.A.) to form LINC01133-WT (sequence: 5′-AGUGCCUCUGAGUUUGUUUACU-3′) and LINC01133-MUT (sequence: 5′-AGUGCCUCUUGAUUGAACCGAU-3′). AC16 cells were cultured in a 96-well plate at 5 × 10^3^ cells/well and were co-transfected with LINC01133-WT and LINC01133-MUT as well as miR-30a-5p mimic (sequence: 3′-GAAGGUCAGCUCCUACAAAUGU-5′) or mimic control using Lipofectamine 3000 Transfection reagent (Thermo Fisher Scientific, U.S.A.) at 37°C. Cells were harvested 48 h post transfection, and then luciferase detection was conducted in dual-luciferase reporter assay system (E1910; Promega, U.S.A.) based on the manufacturer’s instructions. The firefly luciferase activity was normalized to *Renilla* luciferase activity.

### Statistical analysis

All experiments were performed at least three times independently. Results were expressed as mean ± standard deviation (SD). Statistical analysis was conducted using SPSS 19.0 software (IBM Corporation, Armonk, NY, U.S.A.). One-way ANOVA followed by Dunnett’s post hoc test were used to analyze statistical significance. *P*<0.05 was considered statistically significant.

## Results

### Sevoflurane pretreatment improved viability, ameliorated injury and reduced apoptosis in H/R-treated cells

**Figure 1:**
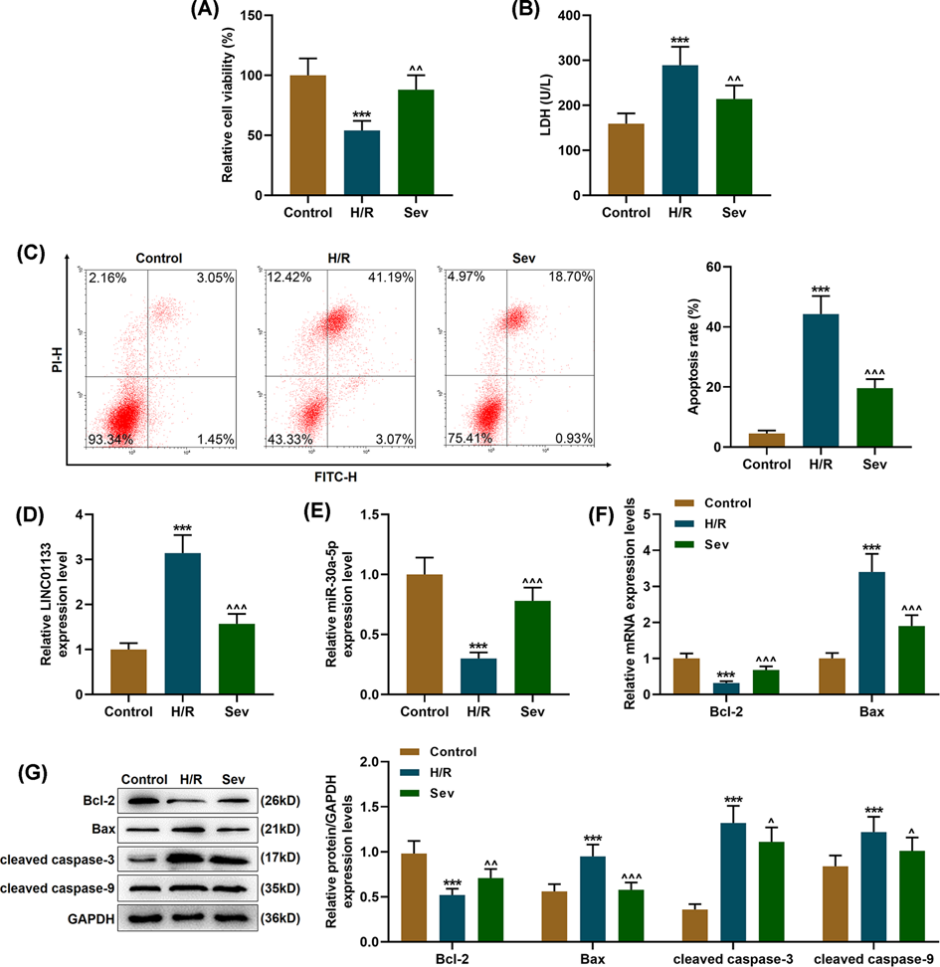
Sevoflurane pretreatment improved viability, ameliorated injury, reduced apoptosis, upregulated miR-30a-5p expression yet downregulated LINC01133 expression in H/R-treated cells (**A**) Relative cell viability after H/R injury and Sev treatment was measured using MTT assay. (**B**) Lactate dehydrogenase (LDH) content in cells after H/R injury and Sev pretreatment was measured with an LDH Detection Kit. (**C**) Cell apoptosis rate after H/R injury and Sev pretreatment was measured by flow cytometry. (**D** and** E**) Relative expressions of LINC01133 (**D**) and miR-30a-5p (E) in cells after H/R injury and Sev pretreatment were detected by quantitative real-time polymerase chain reaction (qRT-PCR). GAPDH (for LINC01133) and U6 (for miR-30a-5p) were used as internal references. (**F**) Relative mRNA expressions of Bcl-2 and Bax in cells after H/R injury and Sev pretreatment were measured by qRT-PCR. GAPDH was used as an internal reference. (**G**) Relative protein expressions of Bcl-2, Bax, cleaved caspase-3 and cleaved caspase-9 in cells after H/R injury and Sev pretreatment were measured by Western blot. GAPDH was used as an internal reference. ***P<0.001, vs. Control; ^∧^*P*<0.05, ^∧∧^*P*<0.01, ^∧∧∧^*P*<0.001, vs. H/R. Sev: Sevoflurane; H/R: hypoxia/reperfusion; LncRNA LINC01133: long non-coding RNA long intergenic non-protein coding RNA 01133.

To discover the possible effects of Sev pretreatment on AC16 cells, we treated cells with Sev after H/R treatment. We first detected cell viability with MTT reagent. As presented in Figure 1A, cell viability dropped after H/R treatment (*P*<0.001, vs. Control). However, an ameliorating effect on cell viability was found after the cells were treated with Sev (*P*<0.01, vs. H/R), which suggested that Sev improved cell viability. LDH leakage has been found as a cellular marker for cell injury for plasma membrane disruption [19], and thus we then examined LDH leakage in cells using an LDH Detection Kit. As Figure 1B exhibits, LDH leakage in cells was up-regulated after H/R treatment (*P*<0.001, vs. Control), whereas Sev treatment resulted in a reduction in LDH leakage in the cells (*P*<0.01, vs. H/R), indicating that Sev ameliorated injury in cells.

In the end, we detected the apoptosis of H/R-induced cells using flow cytometry to discover the effects of Sev on cell apoptosis. In Figure 1C, we discovered that the cell apoptosis rate after H/R treatment was significantly up-regulated (*P*<0.001, vs. Control), while an opposite result was found after we treated the cells with Sev (*P*<0.001), which means that Sev reduced cell apoptosis.

### Sevoflurane pretreatment up-regulated miR-30a-5p expression yet down-regulated LINC01133 expression in H/R-treated cells

To discover the potential effects of LINC01133 and miR-30a-5p on H/R-treated cells, we measured the expressions of LINC01133 and miR-30a-5p in AC16 cells by qRT-PCR. It was found that after H/R treatment, LINC01133 expression was up-regulated yet miR-30a-5p expression was down-regulated in the cells (Figure 1D,E, *P*<0.001, vs. Control). On the contrary, Sev treatment led to down-regulated LINC011CC expression and up-regulated miR-30a-5p expression (Figure 1D,E, *P*<0.001, vs. Control).

### Sevoflurane pretreatment regulated the expressions of apoptosis-related proteins in H/R-treated cells

Since Bax, Bcl-2, cleaved caspase-3, and cleaved caspase-9 have been found to be implicated in cell apoptosis [20,21], we then quantified their expressions by qRT-PCR and Western blot as needed in order to further confirm the molecular mechanisms related to cell apoptosis. In Figure 1F,G, it was observed that Bcl-2 expression were down-regulated whereas expressions of Bax and protein expressions of cleaved-caspase-3 and cleaved-caspase-9 were up-regulated in H/R-treated cells (*P*<0.001, vs. Control); however, Sev pretreatment reversed these expression trends (*P*<0.001, vs. H/R).

### Sevoflurane pretreatment reversed the effect of LINC01133 on LINC01133 and miR-30a-5p expressions in H/R-treated cells

**Figure 2:**
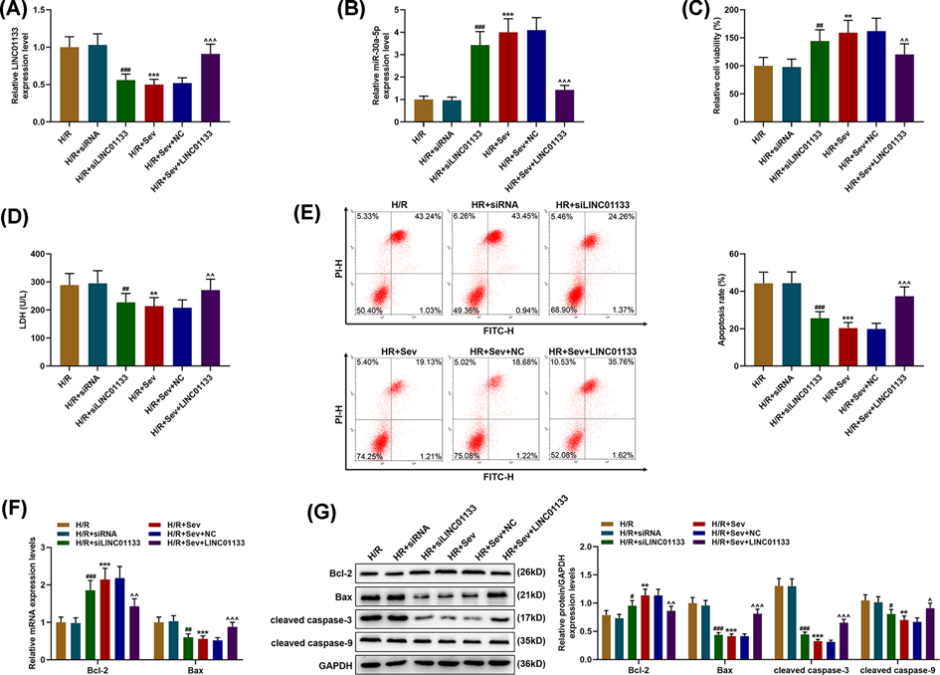
LINC01133 overexpression reversed the effects of Sev on LINC01133 and miR-30a-5p expressions and LDH leakage as well as the viability and apoptosis of H/R-treated cells (**A** and **B**) Relative expressions of LINC01133 (A) and miR-30a-5p (B) in H/R-treated cells after transfection of small interfering RNA for LINC01133 (siLINC01133) and LINC01133 overexpression plasmids as well as Sev pretreatment were detected by qRT-PCR. GAPDH (for LINC01133) and U6 (for miR-30a-5p) were used as internal references. (**C**) Relative viability of H/R-treated cells after transfection of siLINC01133 and LINC01133 overexpression plasmids as well as Sev pretreatment was measured using MTT assay. (**D**) LDH leakage in H/R-treated cells after transfection of siLINC01133 and LINC01133 overexpression plasmids as well as Sev pretreatment was measured with an LDH Detection Kit. (**E**) The apoptosis rate of H/R-treated cells after transfection of siLINC01133 and LINC01133 overexpression plasmids as well as Sev pretreatment was measured using flow cytometry. (**F**) Relative mRNA expressions of Bcl-2 and Bax in cells after transfection of siLINC01133 and LINC01133 overexpression plasmids as well as Sev pretreatment were measured by qRT-PCR. GAPDH was used as an internal reference. (**G**) Relative protein expressions of Bcl-2, Bax, cleaved caspase-3 and cleaved caspase-9 in cells after transfection of siLINC01133 and LINC01133 overexpression plasmids as well as Sev pretreatment were measured by Western blot. GAPDH was used as an internal reference. ^#^*P*<0.05, ^###^*P*<0.001, vs. H/R+siRNA; *P<0.05, **P<0.01, ***P<0.001, vs. Control; ^∧^*P*<0.05, ^∧∧^*P*<0.01, ^∧∧∧^*P*<0.001, vs. H/R. siRNA: small interfering RNA; LDH: Lactate dehydrogenase.

For discovering the effects of Sev and LINC01133 in cells, small interfering RNA for LINC01133 (siLINC01133) and LINC01133 overexpression plasmids were transfected into cells after H/R and Sev pretreatment. In Figure 2A,B, it was shown that after siLINC01133 was transfected into H/R-treated cells, LINC01133 expression was down-regulated yet miR-30a-5p expression was up-regulated (*P*<0.001, vs. H/R). However, after up-regulating LINC01133 expression in the cells, an opposite result was obtained (*P*<0.001, vs. H/R+Sev+NC, Figure 2B), suggesting that LINC01133 could reverse the effects of Sev pretreatment on LINC01133 and miR-30a-5p expressions in H/R-treated cells.

### Sevoflurane pretreatment reversed the effects of LINC01133 on cell viability, cellular injury and apoptosis in H/R-treated cells

To further uncover the role of LINC01133 in cells, we assessed the effects of overexpressed and down-regulated LINC01133 on the viability, injury and apoptosis of H/R-treated cells. As presented in Figure 2C–E, after LINC01133 was down-regulated, cell viability was increased, accompanied by reduced LDH leakage and a lowered apoptosis rate (*P*<0.001, vs. H/R). Conversely, LINC01133 overexpression resulted in reduced cell viability as well as increased LDH leakage and an raised apoptosis rate in H/R-treated cells (Figure 2C–E, *P*<0.001, vs. H/R+Sev+NC).

### Sevoflurane pretreatment could reverse the effects of LINC01133 on the expressions of apoptosis-related proteins in H/R-treated cells

We subsequently measured the expressions of apoptosis-related proteins (Bcl-2, Bax, cleaved caspase-3, and cleaved caspase-9) in H/R-treated cells to confirm the role of LINC01133 in the apoptosis of H/R-treated cells. Data in Figure 2F,G showed that the mRNA and protein expressions of Bcl-2 were up-regulated whereas those of Bax, cleaved-caspase-3 and cleaved-caspase-9 were decreased in H/R-treated cells after transfection of siLINC01133 (*P*<0.001, vs. Control). However, LINC01133 overexpression was found to reverse these trends (Figure 2F,G, *P*<0.001, vs. H/R+Sev+NC).

### MiR-30a-5p was the target of LINC01133

**Figure 3:**
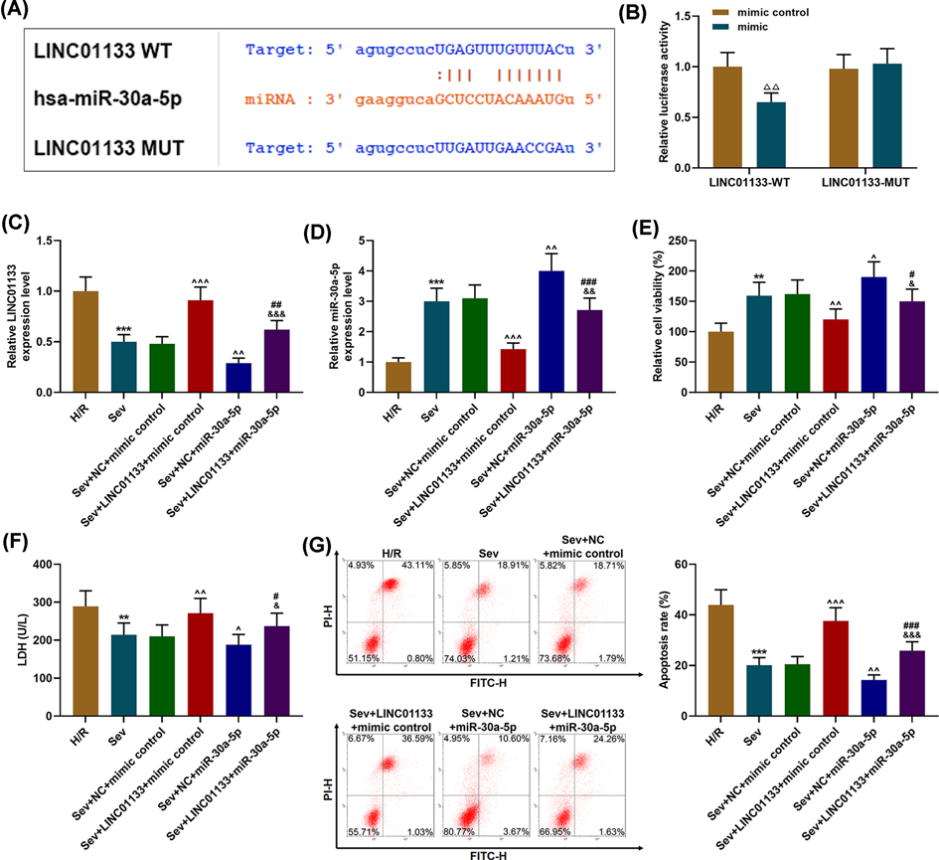
MiR-30a-5p was the target of LINC01133, and LINC01133 overexpression reversed the effects of miR-30a-5p on cell viability, LDH leakage and the apoptosis rate of Sev-pretreated and H/R-treated cells (**A**) Sequences of wild-type LINC01133 (LINC01133-WT; top), hsa-miR-30a-5p (middle), and mutated LINC01133 (LINC01133-MUT; bottom) were provided by Starbase. (**B**) Dual-luciferase reporter assay confirmed that miR-30a-5p was the target of LINC01133. (**C** and **D**) Cells were divided into the following groups: H/R, Sev, Sevolurane+Negative Control+mimic control (Sev+NC+mimic control), Sevolurane+LINC01133+mimic control, Sevolurane+Negative Control+miR-30a-5p (Sev+NC+miR-30a-5p) and Sevolurane+LINC01133+miR-30a-5p. Relative expressions of LINC01133 (C) and miR-30a-5p (D) in cells were measured by qRT-PCR. GAPDH (for LINC01133) and U6 (for miR-30a-5p) were used as internal references. (**E**) Cells were divided into different treatment groups: H/R group, Sev group, Sev+NC+mimic control group, Sevolurane+LINC01133+mimic control group, Sev+NC+miR-30a-5p group and Sevolurane+LINC01133+miR-30a-5p group. Relative cell viability was measured by MTT assay. (**F**) LDH leakage in the cells of different treatment groups was measured with an LDH detection kit. (**G**) Relative cell apoptosis rates of different treatment groups were measured by flow cytometry. ^△△^*P*<0.01, vs. mimic control; **P<0.01, ***P<0.001, vs. H/R; ^∧^*P*<0.05, ^∧∧^*P*<0.01, ^∧∧∧^*P*<0.001, vs. Sev+NC+mimic control; ^#^*P*<0.05, ^##^*P*<0.01, ^###^*P*<0.001, vs. Sev+LINC01133+mimic control; ^&^*P*<0.05, ^&&&^*P*<0.001, vs. Sev+NC+miR-30a-5p.

We predicted the target gene of LINC0133 in our experiments using Starbase, and recognized miR-30a-5p as the target of LINC0133, with their conserved binding sites presented in Figure 3A. To further confirm that miR-30a-5p could bind to LINC01133, we built a luciferase reporter vector which contained their 3′-untranslated regions (3′-UTR). The results of dual-luciferase reporter assay in Figure 3B exhibited that the relative luciferase activity in the LINC01133-WT-mimic group was decreased in comparison with the LINC01133-WT-mimic control group (*P*<0.01), whereas that in the LINC01133-MUT-mimic group was not affected, indicating that miR-30a-5p was the target of LINC01133.

### Effects of LINC01133/miR-30a-5p axis on LINC01133 and miR-30a-5p expressions in Sev-pretreated H/R cells

In this phase, we measured LINC01133 and miR-30a-5p expressions in Sev-pretreated cells after transfection of miR-30a-5p mimic and mimic control. It was found in Figure 3C,D that in the Sev group, the expression of LINC01133 was down-regulated yet that of miR-30a-5p was up-regulated (*P*<0.001, vs. H/R group), whereas up-regulating LINC011CC expression caused an opposite result (*P*<0.001, vs. Sev+NC+ mimic control). Furthermore, after miR-30a-5p was overexpressed in Sev-pretreated cells, the expression of LINC01133 was decreased yet that of miR-30a-5p was up-regulated, whilst LINC01133 overexpression reversed the effects of miR-30a-5p mimic on LINC01133 and miR-30a-5p expressions (Figure 3C,D, *P*<0.001).

### Effect of LINC01133/miR-30a-5p axis on the viability, cellular injury and apoptosis of Sev-pretreated H/R cells

Then we measured cell viability, LDH leakage and the apoptosis rate of Sev-pretreated cells which had been transfected with miR-30a-5p mimic and miR-30a-5p mimic control. It was found that after Sev pretreatment, cell viability was increased, accompanied by decreases in LDH leakage and the cell apoptosis rate, compared with the H/R group (Figure 3E–G, *P*<0.001). However, up-regulation of LINC01133 exerted an opposite effect on the Sev-pretreated cells compared with the Sev+NC+mimic control group (Figure 3E–G, *P*<0.001). In addition, as shown in Figure 3E–G, cell viability was increased yet LDH leakage and the cell apoptosis rate were decreased after up-regulating miR-30a-5p in Sev-pretreated cells, while the effects were reversed by LINC01133 (Figure 3E–G, *P*<0.001).

### Effect of LINC01133/miR-30a-5p axis on the expressions of apoptosis-related proteins in Sev-pretreated H/R cells

**Figure 4:**
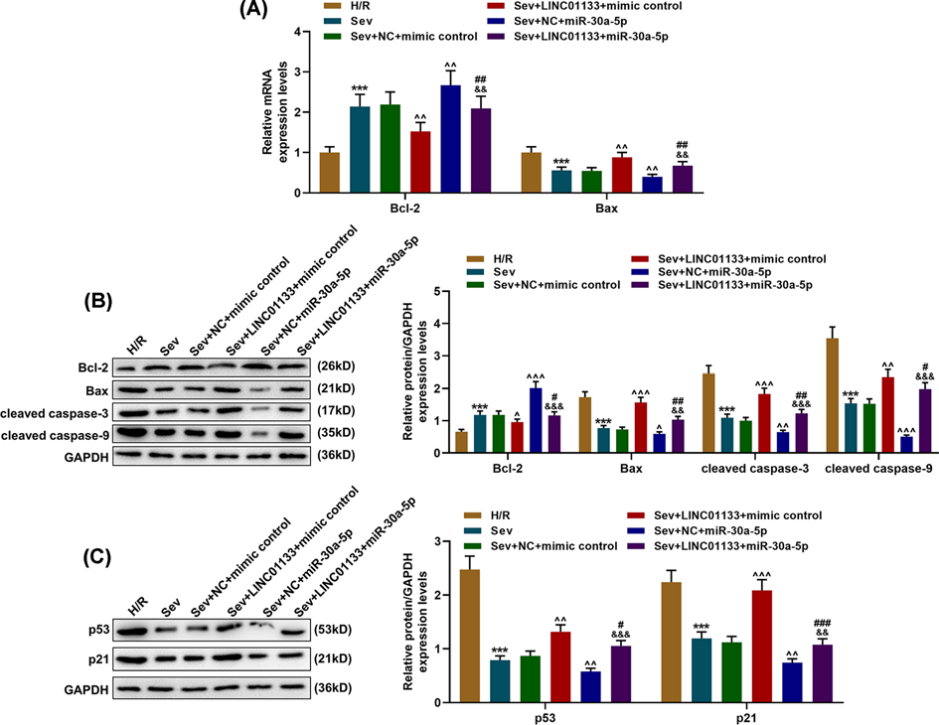
LINC01133 overexpression reversed the effects of miR-30a-5p up-regulation on the expressions of apoptosis-related proteins in Sev-pretreated and H/R-treated cells (**A**) Relative mRNA expressions of Bcl-2 and Bax in the cells of the H/R, Sev, Sev+NC+mimic control, Sevolurane+LINC01133+mimic control, Sev+NC+miR-30a-5p and Sevolurane+LINC01133+miR-30a-5p groups were measured by qRT-PCR. GAPDH was used as an internal reference. (**B**) Cells were divided into different treatment groups: H/R group, Sev group, Sev+NC+mimic control group, Sevolurane+LINC01133+mimic control group, Sev+NC+miR-30a-5p group and Sevolurane+LINC01133+miR-30a-5p group. Relative protein expressions of Bcl-2, Bax, cleaved caspase-3 and cleaved caspase-9 in cells of different treatment groups were measured by Western blot. GAPDH was used as an internal reference. (**C**) Relative protein/GAPDH expressions of p53 and p21 in cells of different treatment groups were measured by Western blot. GAPDH was used as an internal reference. **P<0.01, ***P<0.001, vs. H/R; ^∧^*P*<0.05, ^∧∧^*P*<0.01, ^∧∧∧^*P*<0.001, vs. Sev+NC+mimic control; ^#^*P*<0.05, ^##^*P*<0.01, ^###^*P*<0.001, vs. Sev+LINC01133+mimic control; ^&&^*P*<0.01, ^&&&^*P*<0.001, vs. Sev+NC+miR-30a-5p.

To confirm the down-regulatory effect of miR-30a-5p on cell apoptosis, we measured the expressions of apoptosis-related proteins (Bcl-2, Bax, cleaved caspase-3, and cleaved caspase-9) in Sev-pretreated cells. In Figure 4A,B, we discovered that Sev pretreatment increased the expression of Bcl-2 yet down-regulated those of Bax, cleaved caspase-3 and caspase-9 in cells (*P*<0.001, vs. H/R), and miR-30a-5p further enhanced the effects (*P*<0.001, vs. Sev+NC+mimic control). However, up-regulation of LINC01133 led to an opposite result (Figure 4A,B, *P*<0.001).

### Effect of LINC01133/miR-30a-5p axis on the p53/p21 pathway in Sev-pretreated H/R cells

Since p53 has been found as a nuclear transcription factor with pro-apoptosis properties [22], we then measured the protein expressions of p53 and p21 by Western blot to discover the effects of miR-30a-5p up-regulation and Sev treatment on the p53/p21 pathway in H/R-treated cells. It was found in Figure 4C that after Sev pretreatment, both the protein expressions of p53 and p21 were down-regulated (*P*<0.001, vs. H/R), which were further decreased by miR-30a-5p (*P*<0.01, vs. Sev+NC+mimic control). However, up-regulating LINC01133 posed an up-regulatory effect on these protein expressions (Figure 4C, *P*<0.001).

## Discussion

As one of the commonly used inhalation anesthetics, Sevoflurane (Sev) has been discovered as an important agent with cardioprotective effects [23]. I/R injury in patients has been discovered to be closely related to H/R process, and previous studies uncovered that Sev pretreatment could suppress H/R injury [24,25]. However, the detailed underlying mechanisms of Sev in ameliorating H/R-induced injury have been inadequately discovered and discussed.

In our present study, H/R-induced cardiomyocytes cultured *in vitro* were used to simulate myocardial I/R injury, and with the aim to determine the effects of Sev pretreatment on H/R-induced cardiomyocyte injury, the viability and apoptosis of cardiomyocytes cultured *in vitro* were analyzed. It was found that cell viability was reduced yet the apoptosis rate was increased in the H/R group compared with the Control group, while an opposite result was obtained in the Sev group in comparison to the H/R group, which suggested that Sev pretreatment may up-regulate the viability and reduce the apoptosis of cardiomyocytes after H/R injury.

Myocardial enzyme LDH is one of the common clinical biochemical indicators which can be used to evaluate the degree of myocardial injury [26]. Under physiological conditions, the extracellular LDH level is extremely low; however, after myocardial I/R injury, LDH can be released from cells into the culture medium, which could reflect the degree of myocardial cell damage [27]. In our present study, we discovered that LDH leakage was increased in the cells in the H/R group, whereas that in the Sev group was decreased, indicating that H/R may result in aggravating the injury of cardiomyocytes, while Sev pretreatment could ameliorate myocardial H/R injury, which was consistent with previous study [27].

Mitochondrial apoptosis has been recognized as an important mechanism for H/R-induced damage [28], and H/R simulation may have effects on cell energy metabolism and mitochondrial function, and may cause the release of cytochrome *C* from mitochondria into cytoplasm, thereby activating Caspase-3 and inducing cell apoptosis [29]. Bax and Bcl-2 are discovered as the molecules which have the capability to regulate the mitochondrial membrane permeability to cytochrome *C* [30], while Caspase-9 has been found to play a central role in cell death [31]. Besides, the p53/p21 complex has been found to regulate cell apoptosis [32]. In our study, we measured the expressions of these genes and found the H/R group displayed lower Bcl-2 expression and higher Bax, Cleaved Caspase-3, Cleaved Caspase-9, p53 and p21 expressions than the Control group. The results mean that Sev pretreatment may exert regulatory effects on cell apoptosis so as to protect cardiomyocytes against H/R injury.

Nowadays, the detailed molecular mechanisms by which Sev pretreatment ameliorates myocardial I/R injury in rats and *in vitro* cultured cardiomyocytes remain obscure [33]. Non-coding RNAs (ncRNAs) have been found to play a role in myocardial I/R injury [34,35], but the roles of LINC01133 and miR-30a-5p in myocardial I/R injury are barely discussed. Hence, in order to determine their roles in the amelioration of myocardial I/R injury by Sev pretreatment, we further measured their expressions in cardiomyocytes. In our present study, to our best knowledge, we for the first time showed that Sev pretreatment could reverse the effects of H/R on up-regulating LINC01133 expression and down-regulating miR-30a-5p expression in cardiomyocytes, suggesting that Sev could protect cells against H/R injury via the LINC01133/miR-30a-5p axis. Also, LINC01133 silencing up-regulated cell viability and down-regulated the apoptosis rate when compared with the H/R group, which was similar to the effect of Sev. However, LINC01133 overexpression resulted in down-regulated cell viability and an up-regulated apoptosis rate, which was opposite to the effect of Sev.

Previous studies suggested that miR-30a plays a role in cardiomyocyte injury [36]. In our study, after up-regulating miR-30a-5p in the Sev-pretreated cells, cell viability was ameliorated, the degree of cell injury was decreased, and the cell apoptosis rate was lowered, suggesting that miR-30a-5p could enhance the effects of Sev pretreatment on cardiomyocytes.

According to literature, lncRNA may act as a competing endogenous RNA (ceRNA) of miRNAs via binding to their 3′-UTRs [37]. In our study, with the help of Starbase, we found that LINC01133 could bind to miR-30a-5p. Then we evaluated the effects of up-regulating LINC01133 expression in cardiomyocytes to discover the effects of LINC01133 on the promoting effects of miR-30a-5p on Sev-pretreated cardiomyocytes. We found that up-regulating LINC01133 reversed the promoting effects of miR-30a-5p on Sev-pretreated cardiomyocytes, given that it decreased cell viability as well as increased the degree of cell injury and the cell apoptosis rate, indicating that LINC01133 attenuates the protective effect of Sev-pretreatment on cardiomyocytes against I/R injury through down-regulating miR-30a-5p.

Taken together, it was pointed out in our study that Sev pretreatment could protect cardiomyocytes against H/R-injury via the LINC01133/miR-30a-5p axis. The results in our study provide a novel role of Sev in H/R-injured cardiomyocytes as well as a novel therapeutic target for cardiovascular I/R injury.

## Data Availability

The target gene of miR-30a-5p and their potential binding sites were predicted using Starbase (http://www.starbase.sysu.edu.cn), which were subsequently confirmed using dual-luciferase reporter assay. The analyzed data sets generated during the study are available from the corresponding author on reasonable request.

## Abbreviations

ceRNA: competing endogenous RNA
H/R: hypoxia/reperfusion
I/R: ischemic/reperfusion
LDH: lactate dehydrogenase
ncRNA: non-coding RNA
qRT-PCR: quantitative real-time polymerase chain reaction
Sev: Sevoflurane
